# Low-Temperature Synthesis of Solution Processable Carbon Nitride Polymers

**DOI:** 10.3390/molecules26061646

**Published:** 2021-03-16

**Authors:** Junyi Li, Neeta Karjule, Jiani Qin, Ying Wang, Jesús Barrio, Menny Shalom

**Affiliations:** 1 Department of Chemistry and Ilse Katz Institute for Nanoscale Science and Technology, Ben-Gurion University of the Negev, Beer-Sheva 8410501, Israel; lij@post.bgu.ac.il (J.L.); karjule@post.bgu.ac.il (N.K.); qin@post.bgu.ac.il (J.Q.); wangyin@post.bgu.ac.il (Y.W.); 2 Department of Materials, Royal School of Mines, Imperial College London, London SW72AZ, UK

**Keywords:** polymeric carbon nitride, low-temperature synthesis, solution-processable polymers

## Abstract

Carbon nitride materials require high temperatures (>500 °C) for their preparation, which entails substantial energy consumption. Furthermore, the high reaction temperature limits the materials’ processability and the control over their elemental composition. Therefore, alternative synthetic pathways that operate under milder conditions are still very much sought after. In this work, we prepared semiconductive carbon nitride (CN) polymers at low temperatures (300 °C) by carrying out the thermal condensation of triaminopyrimidine and acetoguanamine under a N_2_ atmosphere. These molecules are isomers: they display the same chemical formula but a different spatial distribution of their elements. X-ray photoelectron spectroscopy (XPS) experiments and electrochemical and photophysical characterization confirm that the initial spatial organization strongly determines the chemical composition and electronic structure of the materials, which, thanks to the preservation of functional groups in their surface, display excellent processability in liquid media.

## 1. Introduction

In the last decade, materials based on graphitic carbon nitride (CN) have attracted extensive attention for their potential application as catalysts for various reactions such as water splitting [1,2,3,4], CO_2_ reduction [5,6], organic pollutant degradation [7], and organic transformations [8,9,10] in sensing [11,12,13] and in other fields [14], owing to their photo- and electro-catalytic properties, stability under harsh chemical conditions, easy synthesis, and low cost.

One drawback of polymeric CN is that high temperatures (>500 °C) are required for its synthesis. Moreover, the insolubility of CN in most solvents hinders its further processability and deposition on substrates that cannot stand high temperatures (e.g., plastic, membranes) [15,16]. Synthesizing CN materials at lower temperatures would reduce the needed energy, and therefore, the cost of the final materials. Furthermore, at low reaction temperatures, only partial condensation is achieved; CN polymers of a smaller molecular size are obtained, with preserved functional groups that would be otherwise released at higher calcination temperatures. As a result, the dispersibility, and hence, the solution processability of such CN materials, is expected to be improved.

In recent years, our group and others have shown that the condensation of supramolecular assemblies comprising carbon- and nitrogen-based building blocks with high C/N ratios can lead to the formation of semiconducting polymers with photocatalytic properties that are adapted for the degradation of organic pollutants, water splitting, or CO_2_ reduction [17,18,19,20,21,22]. A major factor in the design of the CN material properties lies in the starting C/N ratio in the reactant(s). However, the spatial arrangement of the atoms within the molecule and its influence on the final optical, structural, and catalytic properties of CN materials has rarely been discussed [23,24,25]. Their processability has also not been studied until now.

In this work, we synthesized semiconducting CN polymers by the thermal condensation of acetoguanamine (AGA) and 2,4,6-triaminopyrimidine (TAP) at various temperatures [26,27]. These organic molecules are structural isomers, i.e., they have the same molecular formula but a different spatial arrangement of their atoms; upon condensation, their polymerization pathway differs, leading to distinct materials with different electronic structures and chemical compositions. This allowed us to correlate the properties of the materials with the initial chemical structure of the reactants and show the potential of said materials in different photoelectrocatalytic scenarios.

## 2. Results

**Figure 1:**
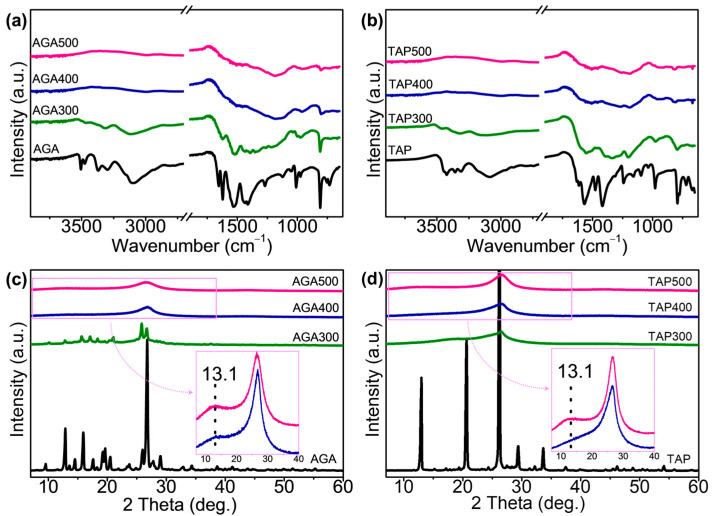
Fourier-transform infrared spectroscopy (FTIR) spectra of (**a**) acetoguanamine (AGA)- and (**b**) 2,4,6-triaminopyrimidine (TAP)-derived materials. X-ray diffraction analysis (XRD) patterns of (**c**) AGA- and (**d**) TAP-derived materials. The inserts in (**c**) and (**d**) are enlarged views of the corresponding area.

While monomers such as TAP and AGA have been shown taking part in melamine-cyanuric acid assemblies for modulating the electronic structure of the resulting CNs, up to now they have not been studied as a single precursor for the preparation of photoactive CN polymers. To do so, TAP and AGA were heated at different temperatures to afford the materials pictured in Figure S1 (AGA300, for example, is the material prepared by the condensation of AGA at 300 °C). Fourier-transform infrared spectroscopy (FTIR) analysis (Figure 1a,b) suggested the formation of polymeric materials. The initial monomers showed bands at 2900–3500 cm^−1^, assigned to N−H stretching; the strong peaks between 1200 and 1700 cm^−1^ were typical of stretching vibrations of triazine rings; the sharp peak at 806 cm^−1^ corresponded to the breathing vibration of the triazine group [28]. At 300 °C, the peaks corresponding to amino groups in AGA300 and TAP300 became broader and weaker, suggesting the occurrence of at least some condensation. Upon calcination at 400 °C, those vibrations vanished, indicating the successful formation of a polymeric structure at 400 °C.

X-ray diffraction analysis (XRD, Figure 1c,d) of the products condensed at 300 °C disclosed that significant changes in the crystal structure already occurred at this temperature. Interestingly, AGA300 showed more diffraction peaks than TAP300, which may be due to the presence of the methyl group outside the triazine ring producing additional periodic structures during the incomplete polymerization [20,29]. AGA400 and TAP400 both exhibited a strong peak at 26.5° and a broad peak at 13.1°, attributed to the (002) and (100) planes of the graphitic structures, respectively. The corresponding (*d*) spacing was 0.335 nm, which matched well with the *d_002_* spacing (0.34 nm) in the crystalline graphitic CN [30].

**Scheme 1:**
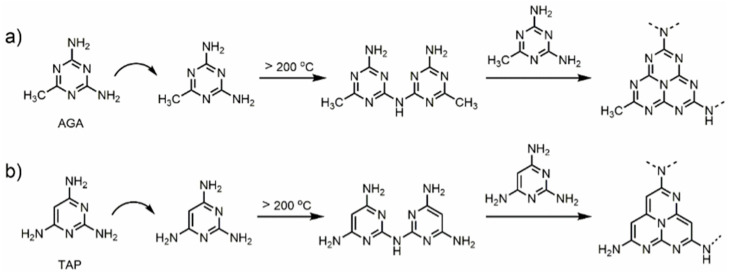
Proposed structure of the condensation intermediates and final products of the thermal condensation of (**a**) acetoguanamine and (**b**) 2,4,6-triaminopyrimidine.

Thermogravimetry analysis (TGA, Figure S2) indicated that both AGA and TAP underwent a dramatic weight loss between 200 and 300 °C, i.e., at a much lower temperature than melamine (400 °C), a typical monomer for the synthesis of polymeric carbon nitride materials (C_3_N_4_H_x_) [23,31]. These observations, along with the FTIR and XRD data, suggest a different polymerization route for these monomers—a route that leads to the formation of CN polymers at lower calcination temperatures than the standard route for carbon nitride materials (Scheme 1).

Scanning electron microscopy and transmission electron microscope (SEM and TEM, Figures S3 and S4) showed that AGA-derived materials presented a granular morphology, with particles that become hollow and porous at higher calcination temperatures. In contrast, TAP-derived materials displayed a smooth homogeneous surface. The size of both the AGA-and TAP-derived materials was significantly reduced compared with their corresponding precursors (Figure S5). N_2_ adsorption–desorption isotherms of AGA- and TAP-derived materials (Figure S6) revealed that the Brunauer–Emmett–Teller (BET) surface area of all these samples was small.

^1^H and ^13^C nuclear magnetic resonance (NMR) experiments were carried out to elucidate the structure of the materials obtained by calcination of the monomers at 300 °C. The ^1^H NMR spectrum of the monomer AGA contained two signals attributable to the primary amine –NH_2_ at 6.65 ppm and the –CH_3_ groups at 2.05 ppm (Figure S7). After the calcination of AGA at 300 °C, the NMR spectrum showed a broadening of the peak at 6.62–6.67 ppm, which suggests the formation of at least some oligomers in AGA300, and the –CH_3_ groups were preserved [32]. The newly formed broad peak at 7.12–7.39 ppm, which belonged to the –NH protons, suggested the formation of linear polymers of heptazine linked by –N(H) groups forming CN sheets (Scheme 1). The spectrum of the other monomer, TAP, displayed three signals: (a) at 4.84 ppm for –CH, (b) at 5.36 ppm for the –NH_2_ in para position to the CH, and (c) at 5.54 ppm for the other two –NH_2_. The peaks in the TAP300 spectrum were shifted downfield and were broader than those observed for its monomer (Figure S8). Additionally, –NH protons of the formed CN sheets showed a downfield shift with a broad signal at 6.01–6.32 ppm. The ^13^C NMR spectra of AGA300 and TAP300, which showed characteristic peaks of the monomeric unit, also confirmed the formation of a polymer with AGA and TAP repeating units (further discussed in Figures S9 and S10).

Overall, the results suggested that both monomers underwent incomplete polymerization at 300 °C, resulting in intermediate structures of CN polymers, as shown in Scheme 1 [33]: the signals for the various –NH_2_ units of these structures were superimposed on one another, causing a broadening and a downfield shift of peaks in the ^1^H NMR spectra. Thanks to the remaining –NH_2_ groups after the partial condensation, the materials were solution-processable in various organic solvents (Figure S11).

X-ray photoelectron spectroscopy (XPS) was carried out to further characterize the chemical states of C and N in AGA- and TAP-derived materials. All the high-resolution C 1s spectra for AGA-derived materials could be deconvoluted in three different chemical binding energies (Figure S12) at 284.8, 286.7, and 287.7 eV, corresponding to sp^2^ C–C within the structure and adventitious carbon, the methyl moiety, and sp^2^-hybridized carbon bonded to nitrogen within the triazine ring (N–C=N), respectively. In the case of the TAP-derived materials, the C 1s spectra were deconvoluted into four peaks (Figure S13) at 284.8, 286.2, 287.2, and 288.2 eV, corresponding to adventitious carbon, C–C=C bonds inside the ring, sp^2^-hybridized carbon bonded to nitrogen in aromatic rings (N–C=N), and C–C=N bonds, respectively [34]. The XPS C 1s spectra of both AGA- and TAP-derived materials remained nearly unchanged at the different calcination temperatures, indicating that the carbon-containing functional groups were largely retained during the polymerization process. The two peaks in the N 1s XPS spectra of AGA300 and TAP300, shown in Figure S14, at 398.5 and 399.5 eV, can be respectively assigned to sp^2^-nitrogen atoms in triazine rings (C–N=C) and amine groups. At 400 °C and 500 °C, the new peak at 400.7 eV can be attributed to the partially hydrogenated N atoms of NH, resulting from the partial condensation of amines. Finally, the peak at 399.7 corresponded to N–(C)_3_ rather than the above neutral amines (at 399.5 eV) since no primary amino group was present according to the FTIR results. The N 1s XPS peaks of AGA and TAP were similar, and both confirmed the condensation phenomenon with increasing calcination temperature.

These results are consistent with the proposed intermediate structures shown in Scheme 1. The C/N mass ratios (Table S1) of AGA- and TAP-derived materials, calculated from the elemental analysis (EA) results, increased gradually with higher calcination temperatures, suggesting an increasing higher condensation degree in line with the FTIR and XRD results. Remarkably, from 400 °C to 500 °C, the C/N mass ratio of AGA500 remained almost unchanged, whereas the C/N mass ratio of TAP500 substantially increased; this may be due to the complete insertion of C=C moieties in a CN-like polymer, replacing the typical triazine units. The comparison of these two isomers demonstrated that the spatial organization of the atoms in the initial CN monomer(s) strongly affects the structure of the final polymer and, therefore, its overall properties.

We further analyze this fact by comparing the condensation of melamine (MA) and 2,4,5,6-tetraaminopyrimidine (TtAP) (structures in Figure S15), which have a similar structure, with one nitrogen from the aromatic ring of MA being substituted by a primary amine-substituted carbon in TtAP. This additional carbon and the higher tendency of amine groups to be released upon thermal condensation resulted in a C-rich material with a condensation temperature as low as 400 °C, while melamine showed features of melem-like moieties [35]. Hence, the position and type of nitrogen are critical, particularly because the NH_2_ groups are more thermally labile and may be eliminated during the condensation, while the nitrogen atoms of the aromatic ring are more likely to remain in the final material after calcination. Further discussion and characterization of these materials can be found in the (Supporting Information Figures S16–S18). Hereafter, we focused our study on the implications of the spatial location of the atoms from AGA and TAP on the final CN materials’ properties.

**Figure 2:**
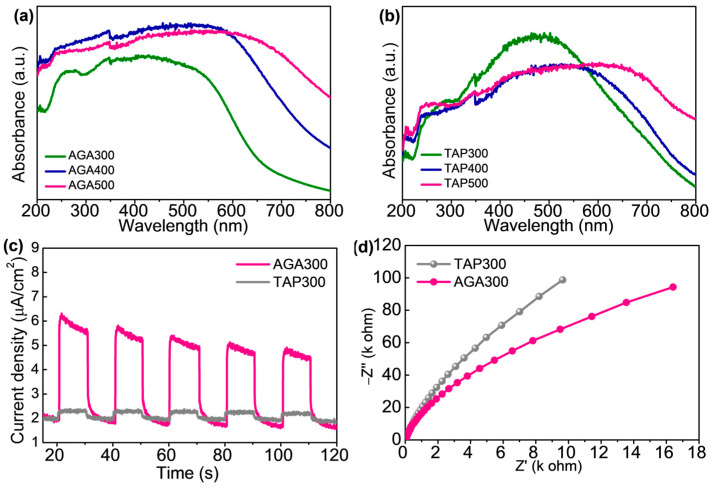
Ultraviolet-visible spectroscopy (UV-vis) absorbance spectra of (**a**) AGA- and (**b**) TAP-derived materials. (**c**) Transient photocurrent responses and (**d**) Nyquist electrochemical impedance spectroscopy (EIS) plots of AGA300 and TAP300.

Ultraviolet-visible spectroscopy (UV-vis) absorption spectroscopy (Figure 2a,b) of the AGA- and TAP-based materials revealed that all samples showed a semiconductor-like behavior; they displayed narrow band gaps compared to standard CN [1], making them particularly suitable for photocatalytic reactions. The absorption band edges of both AGA- and TAP-derived materials showed a red shift upon higher calcination temperatures; this was also reflected by the color of the samples (Figure S1). This red shift was a consequence of the narrowing of the band gap of the materials (Figure S19) at the higher calcination temperatures, owing to further condensation and changes in the C/N ratio.

The processability of CN polymers is usually quite challenging on account of their low solubility in most solvents, which hinders the obtention by solution-based methods of thin films for photoelectronic devices [36,37,38,39]. The processability of the new materials was illustrated by dispersing them in ethanol and drop-casting the suspension on conductive glass to form high-quality films (resulting electrodes in Figure S20). The semiconducting character of the materials obtained at the low calcination temperature resulted in films suitable for photoelectrochemical measurements; we evaluated the charge separation efficiency of photogenerated electron-hole pairs by measuring the generated photocurrent in a standard photoelectrochemical cell [4,40] and by using electrochemical impedance spectroscopy (EIS). As shown in Figure 2c, the photocurrent density of AGA300 was higher than that of TAP300, reaching up to 6 µA cm^−2^. Additionally, the arc radius of the EIS Nyquist plot (Figure 2d) of AGA300 was considerably smaller than that of TAP300, indicating a lower charge transfer resistance. The electrodes of AGA- and TAP-derived materials prepared at a higher calcination temperature were not suitable for the solution-based formation of films: the material peeled off the electrode on contact with the electrolyte. Although the observed photocurrent values were not as high as those reported in the literature for other CN-based materials, to the best of our knowledge, this is the first report showing photocurrent generation from a semiconducting CN polymer prepared at such a low calcination temperature.

**Figure 3:**
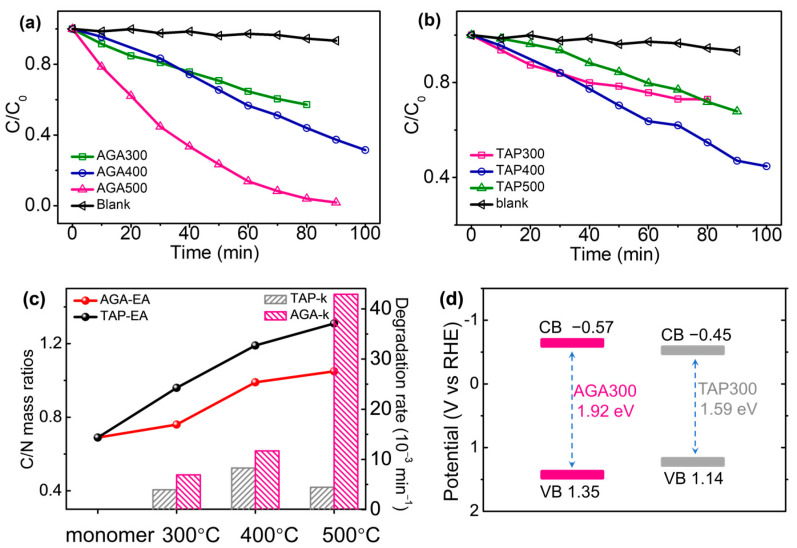
Relative concentration of rhodamine B (RhB) as a function of illumination time for (**a**) AGA- and (**b**) TAP-derived materials. (**c**) C/N mass ratios and kinetic degradation rate *k* as functions of the calcination temperature for AGA- and TAP-derived materials. (**d**) Estimated band structures of AGA300 and TAP300.

We also evaluated the photoactivity of the samples by monitoring their ability to degrade the dye rhodamine B (RhB) under visible light. As shown in Figure 3a,b, both AGA- and TAP-derived materials showed photocatalytic degradation, even those made at a condensation temperature of only 300 °C—a very low calcination temperature compared to that commonly used to synthesize semiconductor CN polymers [14,15]. The photocatalytic degradation reaction kinetics were quantified by fitting the data to a pseudo-first-order kinetic equation (with correlation coefficients of R^2^ > 0.95, Figure S21) [41]. The kinetic degradation rate *k* of AGA materials prepared at various temperatures followed the order of AGA500 (42.9 × 10^−3^ min^−1^) > AGA400 (11.7 × 10^−3^ min^−1^) > AGA300 (6.9 × 10^−3^ min^−1^), in line with the C/N ratios (Figure 3c). In contrast, the *k* rate of TAP-derived materials followed the order TAP 400 (8.3 × 10^−3^ min^−1^) > TAP500 (4.5 × 10^−3^ min^−1^) > TAP300 (3.9 × 10^−3^ min^−1^). We attribute this to the modification of the electronic structure owing to a high C/N ratio in TAP500 (Figure 3c). We hypothesized that, while in AGA-based materials the –CH_3_ groups would act as a functional group in a lattice similar to that of carbon nitride [26], in TAP, the sp^2^ carbon within the pyrimidinic ring would be retained in the structure of the material, substantially enhancing the conductivity of the material, and modifying its semiconducting behavior (Figure 3d); these changes were confirmed by a Tauc plot (Figure S19) and a Mott–Schottky analysis (Figure S22).

## 3. Materials and Methods

### 3.1. Materials Synthesis

Melamine (MA), acetoguanamine (AGA), 2,4,6-triaminopyrimidine (TAP) and 2,4,5,6-tetraaminopyrimidine (TtAP) were used as raw materials without further purification. In a typical reaction, the starting monomer was placed in a ceramic crucible with a lid, then heated to the desired temperature at 4 °C/min and kept at this temperature for 4 h under a N_2_ atmosphere. The samples were then ground and denoted as AGAT (where T is the reaction temperature, T = 300, 400, 500), TAPT (T = 300, 400, 500), TtAPT (T = 300, 400) and MAT (T = 300, 400), respectively.

### 3.2. Materials Characterization

The Fourier-transform infrared spectroscopy (FTIR) spectra were collected from a Thermo Scientific Nicolet iN 10Mx infrared microscope. X-ray diffraction (XRD) patterns were obtained using an Empyrean powder diffractometer (Panalytical). ^1^H and ^13^C NMR spectra were recorded in DMSO-*d*_6_ on 400 MHz NMR spectrometers (Bruker DPX 400). Scanning electron microscopy (SEM) images were taken at 3 kV with a JEOL JSM-7400F system equipped with a Thermo Scientific NORAN System SIX. Ultraviolet-visible spectroscopy (UV-Vis) spectra were recorded using a Cary 100 spectrophotometer. X-ray photoelectron spectroscopy (XPS) was performed on a Thermo Fisher Scientific ESCALAB 250 using monochromated Al Kα X-rays (1486.6 eV). Charging effects were corrected by calibrating the spectra relative to the carbon C1′s peak, positioned at 284.8 eV. Elemental analysis (EA) results were collected using a Thermo Scientific Flash Smart elemental analyzer OEA 2000.

### 3.3. Dye Degradation Experiments

The photocatalytic activity was initially evaluated by measuring the photodegradation of the rhodamine B (RhB) dye under white-light irradiation (Bridge lux BXRA-50 C5300; λ *>* 410 nm). In a typical experiment, an aqueous RhB solution (20 mL, 10 mg L^−1^) and the desired material (20 mg) were mixed in a glass vial in the dark and under continuous stirring until an adsorption–desorption equilibrium was reached. After turning on the light, aliquots were withdrawn from the suspension at regular time intervals. The remaining concentration of the RhB dye was monitored by optical absorption (measured at λ_max_ = 554 nm) and plotted as the normalized concentration *C*/*C_0_*. The photocatalytic degradation reaction kinetics in both cases were quantified by the following pseudo-first-order kinetic Equation (1):

ln (*C*/*C_0_*) = − *k t* (1)

where *k* is the apparent kinetic rate constant and *C_0_* and *C* are the initial concentration at *t* = 0 and instantaneous concentration of RhB solution at irradiation time *t,* respectively.

### 3.4. Electrochemical Measurements

Electrochemical measurements were recorded using a three-electrode system on an Autolab potentiostat (Metrohm, PGSTAT 101). A Pt-foil electrode and an Ag/AgCl (Saturated KCl) electrode were used as the counter and reference electrodes, respectively. Mott–Schottky (1/C^2^ vs. V) measurements were carried out in a 1 M Na_2_SO_4_ aqueous solution as the electrolyte at a frequency of 2.48 kHz solution.

## 4. Conclusions

In summary, we showed the simple synthesis of solution-processable, photoactive CN polymers by the polymerization of acetoguanamine or 2,4,6-triaminopyrimidine at low calcination temperatures (300 and 400 °C). Compared to the standard carbon nitride monomers where the carbons are adjacent to the nitrogen (e.g., melamine, urea, etc.), the presence of a carbon linked to another carbon atom (whether inside or outside the triazine ring) resulted in a decrease of the polymerization temperature. Furthermore, we shed light on the correlation between the spatial organization of the carbon and nitrogen atoms on the final structural, optical, and catalytic properties of the material. The new polymeric materials exhibited good activity as photocatalysts and with extended optical response, owing to their narrow bandgap. This work shines light on critical considerations for the rational design from the molecular level of new, solution-processable carbon- and nitrogen-based photocatalysts and films.

## Supplementary Materials

Supplementary Materials are available online.

## Sample Availability

Samples are available from the authors.
