# Supplementary material for: Low-Temperature Synthesis of Solution Processable Carbon Nitride Polymers
Source: Molecules. 2021 Mar 16;26(6):1646. doi: 10.3390/molecules26061646 (PMC8000294; doi:10.3390/molecules26061646)
Supplement: Supplementary file 1 [file molecules-26-01646-s001.pdf]

Supporting Information

Low-Temperature Synthesis of Processable Semiconductive Carbon Nitride Polymers

Junyi Li,[1] Neeta Karjule,[1] Jiani Qin,[1] Ying Wang,[1] Jesús Barrio[*,1,2] and Menny Shalom[* 1]

1 Department of Chemistry and Ilse Katz Institute for Nanoscale Science and Technology Ben-Gurion University of the Negev, Beer-Sheva 8410501, Israel

E-mail: mennysh@bgu.ac.il

2 Department of Materials, Imperial College London, Royal School of Mines, London SW72AZ, United Kingdom

E-mail: j.barrio-hermida@imperial.ac.uk

*Correspondence: Jesús Barrio: j.barrio-hermida@imperial.ac.uk; Menny Shalom: mennysh@bgu.ac.il

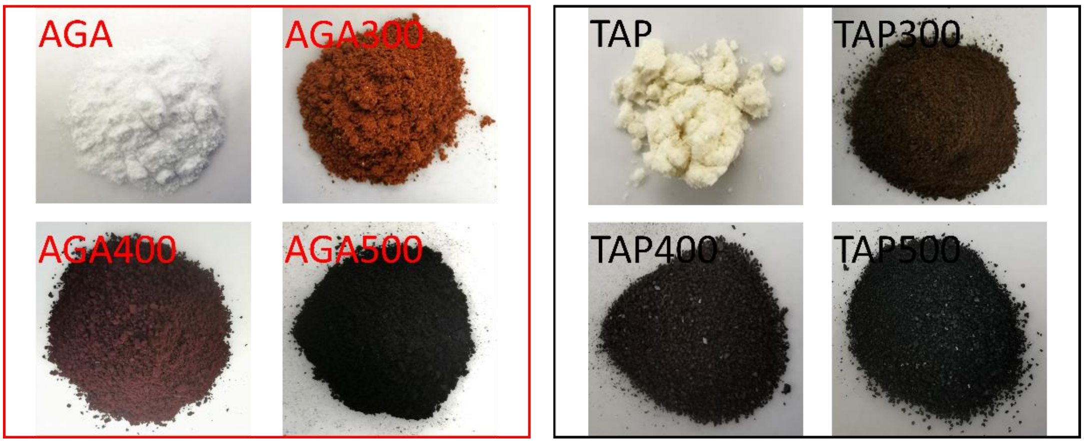

Figure S1. Digital photographs of AGA- and TAP-derived materials. The number (e.g., AGA300) indicates the temperature of the condensation.

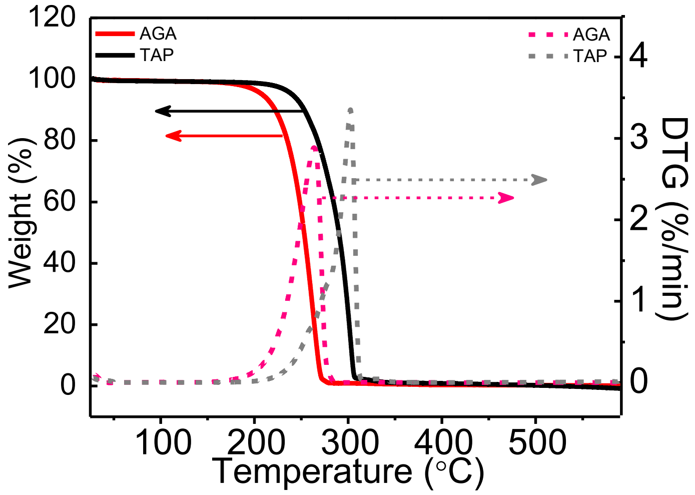

Figure S2. Thermogravimetric analysis of AGA and TAP under N_2 atmosphere.

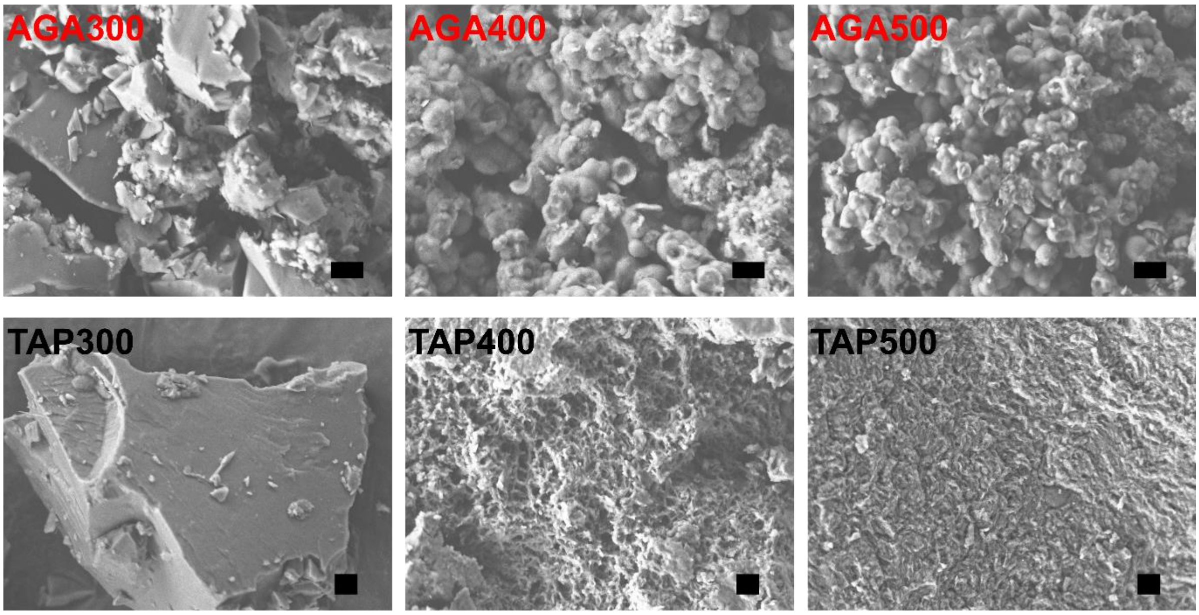

Figure S3. SEM images of AGA- and TAP-derived materials (All black scale bars are 2 μm.) and SEM image of AGA500

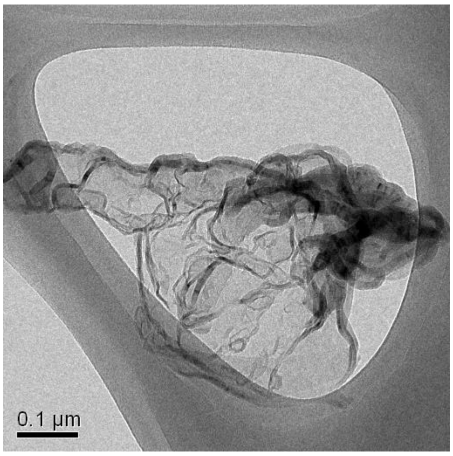

Figure S4. TEM imageof AGA300.

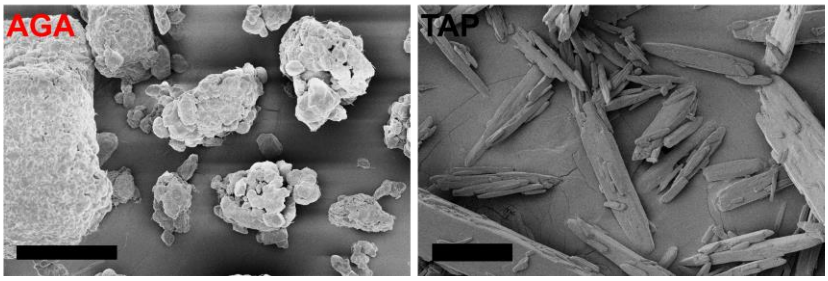

Figure S5. SEM images of AGA and TAP. (All black scale bars are 50 μm.)

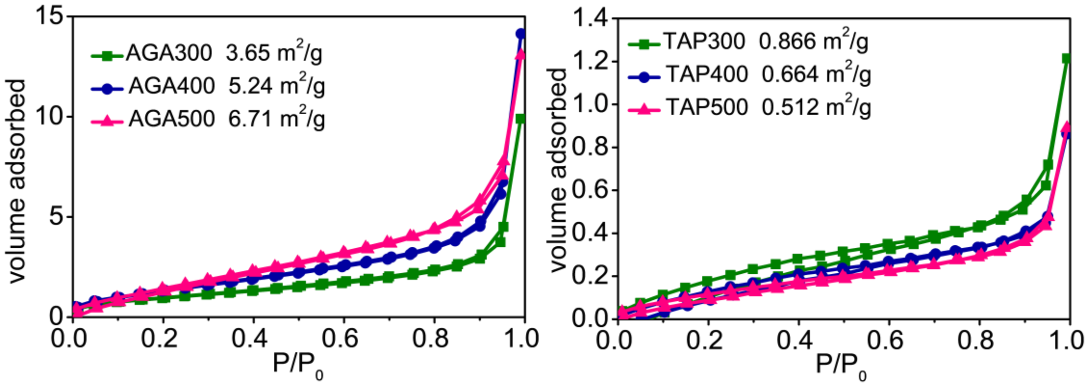

Figure S6. N_2 adsorption-desorption isotherms of AGA- and TAP-derived materials.

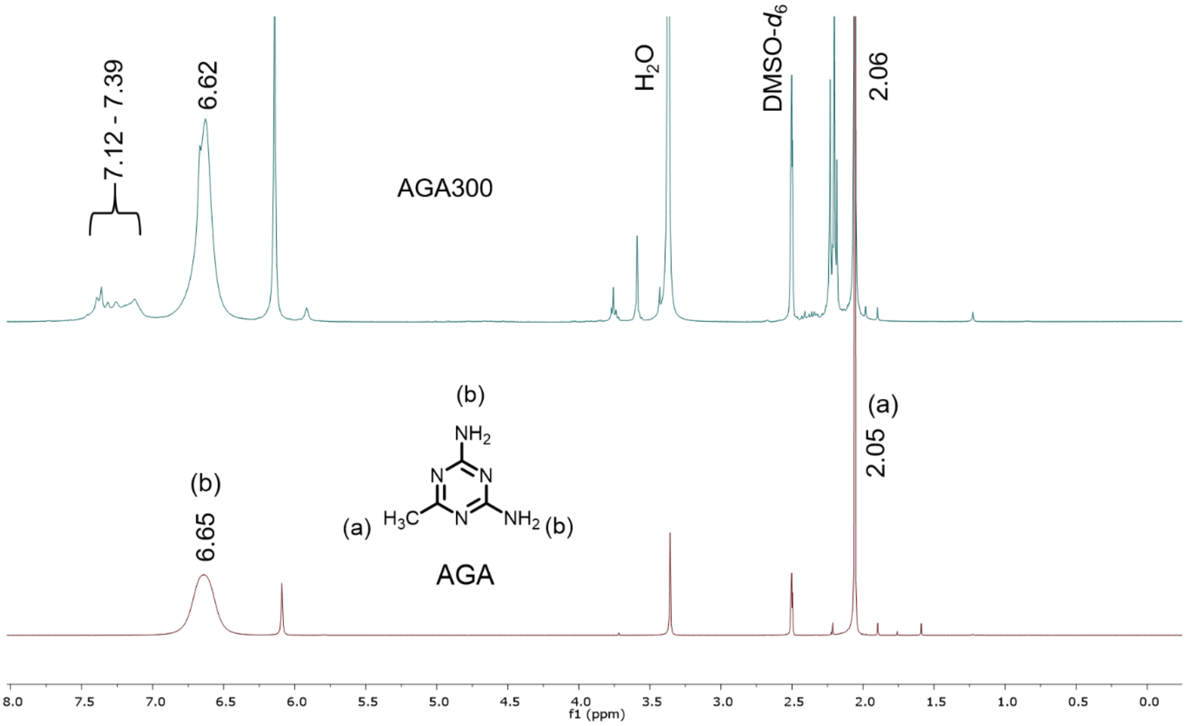

Figure S7. ^{1}H NMR spectra of AGA (brown) and AGA300 (light blue) in DMSO-d_6 at 400 MHz.

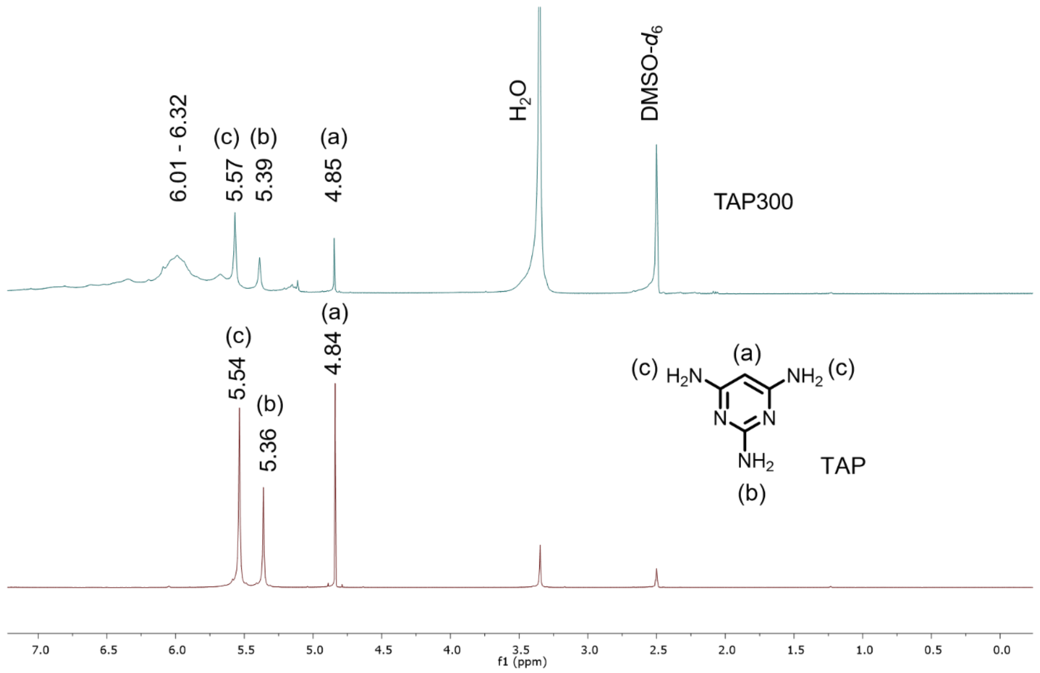

Figure S8. ^{1}H NMR spectra of TAP (brown) and TAP300 (light blue) in DMSO-d_6 at 400 MHz.

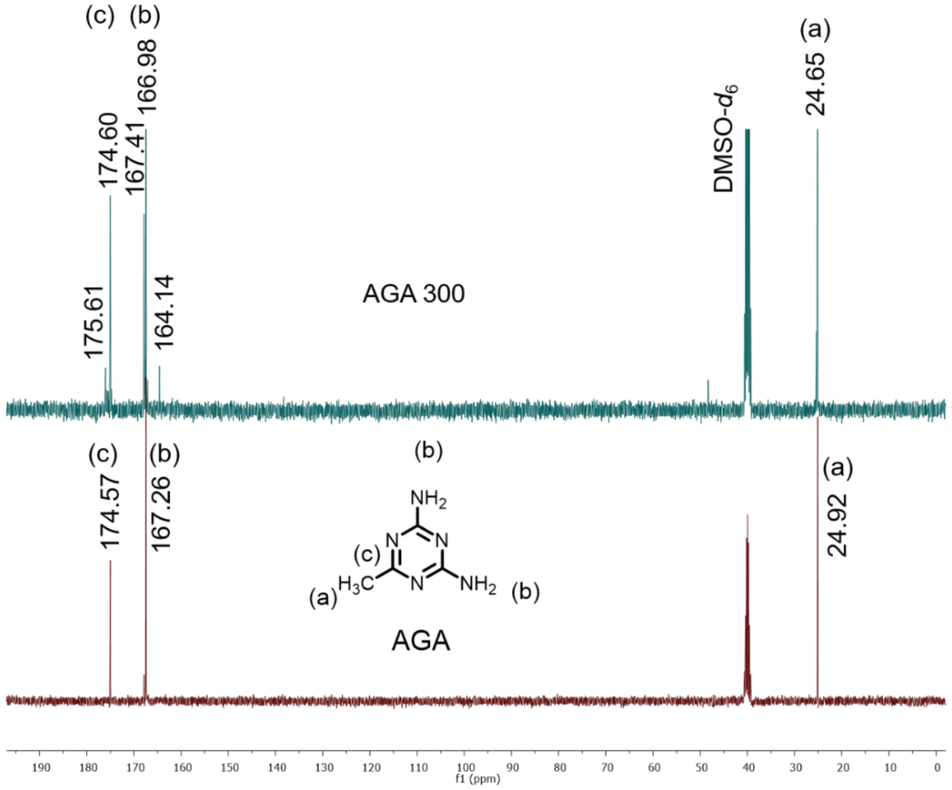

Figure S9. ^{13}C NMR spectra of AGA (brown) and AGA300 (light blue) in DMSO-d_6 at 400 MHz.

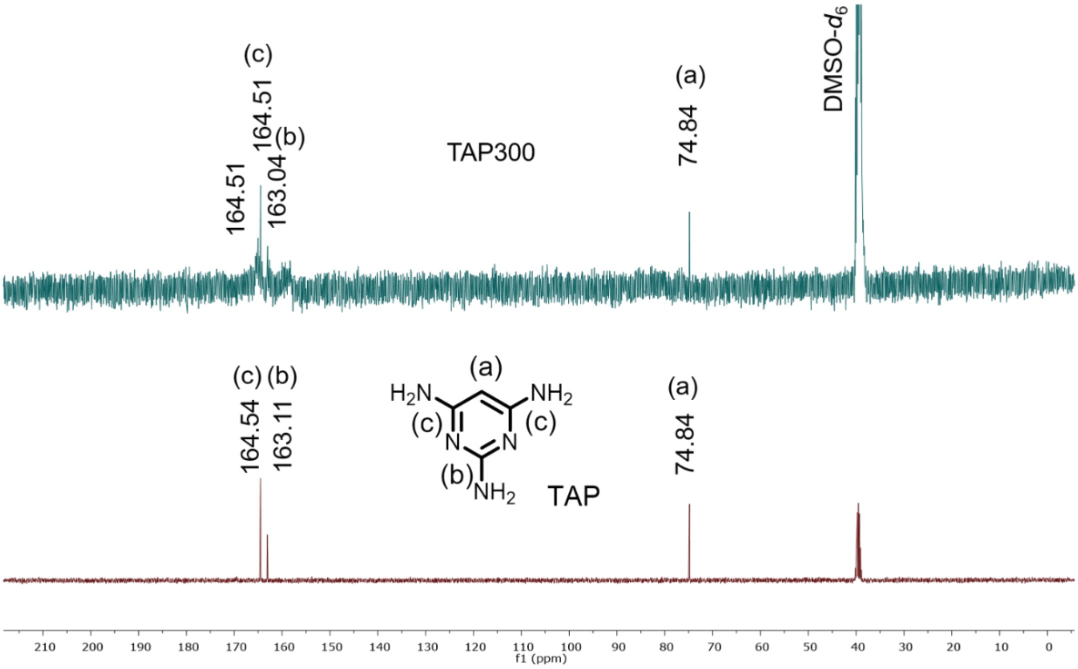

Figure S10. ^{13}C NMR spectra of TAP (brown) and TAP300 (light blue) in DMSO-d_6 at 400 MHz.

In the ^{13}C NMR spectrum, AGA-300 shows new signals at 48.2, 164.14, 166.98 and 175.61 ppm in addition to the characteristic peaks of the monomer (i.e. 24.92, 167.26, and 174.57 ppm), which confirm that the polymer formation has taken place (Figure S9).

The ^{13}C NMR spectrum of TAP-300 consists of characteristic peaks of the monomer unit (74.84, 163.11, 164.54 ppm) that corroborate that the polymer at 300 ^{o}C contains the TAP repeating units (Figure S10).

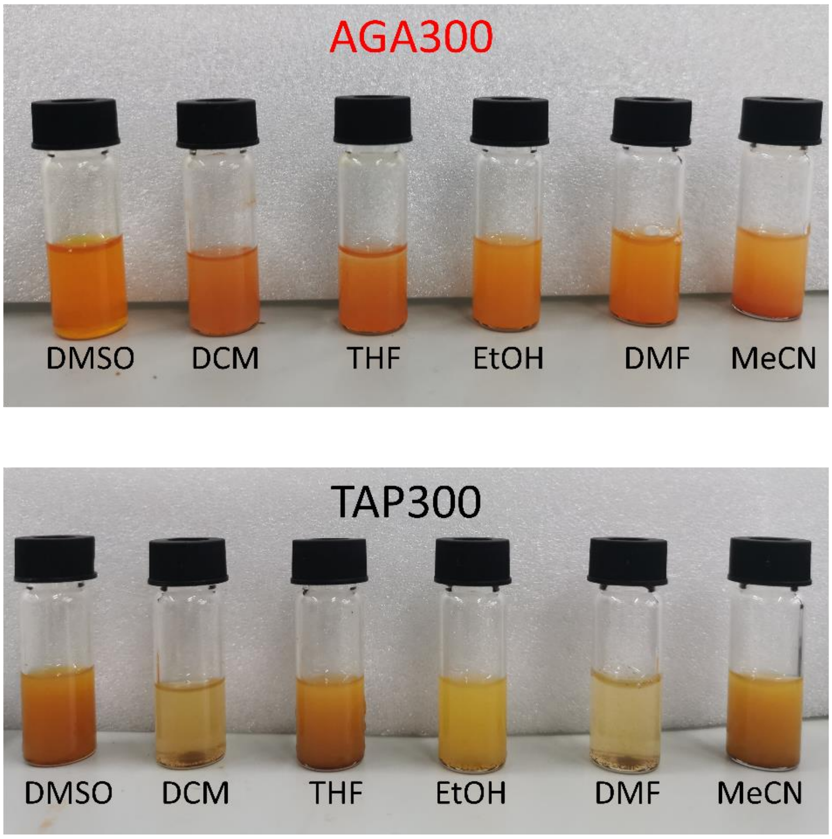

Figure S11. Digital photographs of AGA300 and TAP300 dispersed in different organic solvents: Dimethyl Sulfoxide (DMSO), Dichloromethane (DCM), Tetrahydrofuran (THF), Ethyl alcohol (EtOH), N,N-Dimethylformamide (DMF), and Acetonitrile (MeCN).

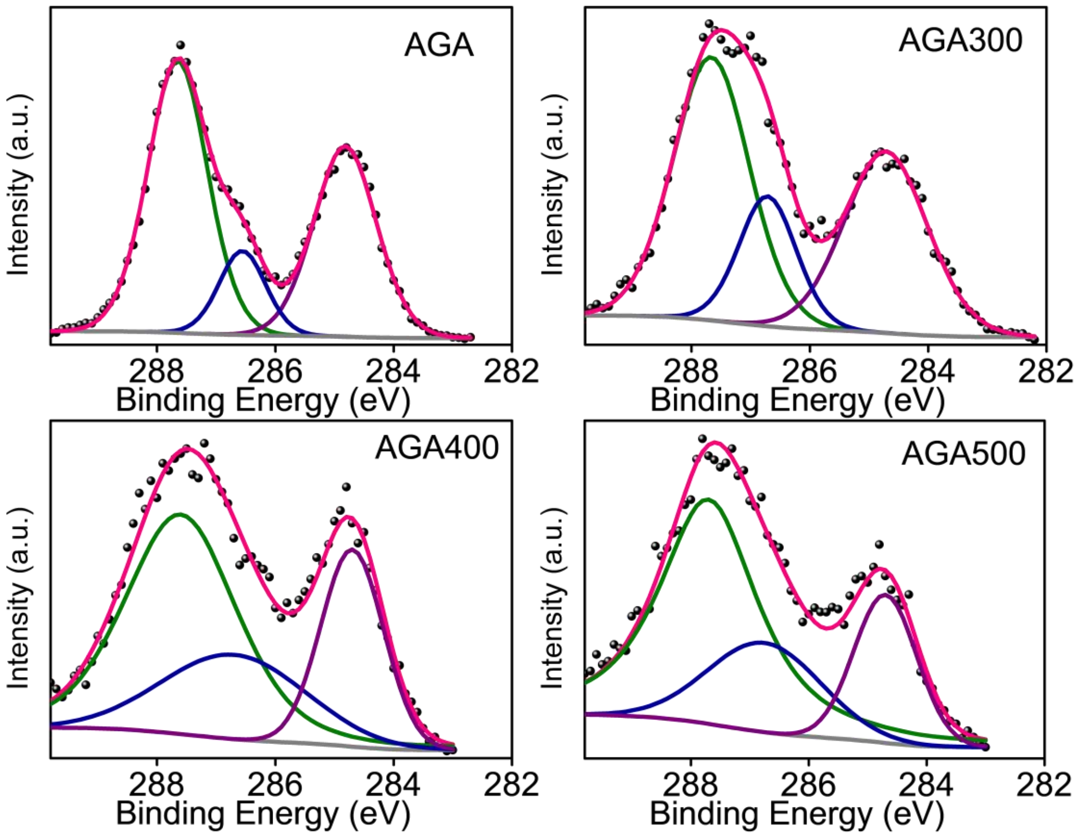

Figure S12. High-resolution C 1s XPS spectra of AGA-derived materials.

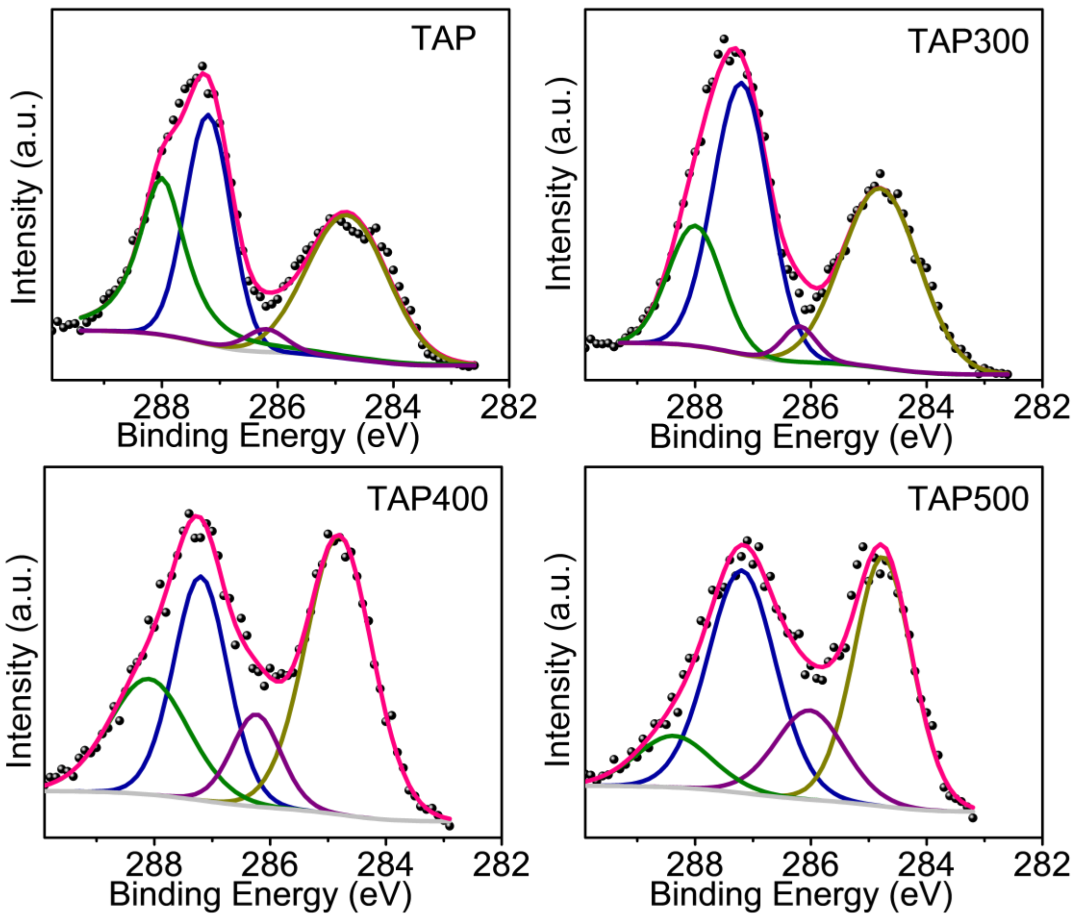

Figure S13. High-resolution C 1s XPS spectra of TAP-derived materials.

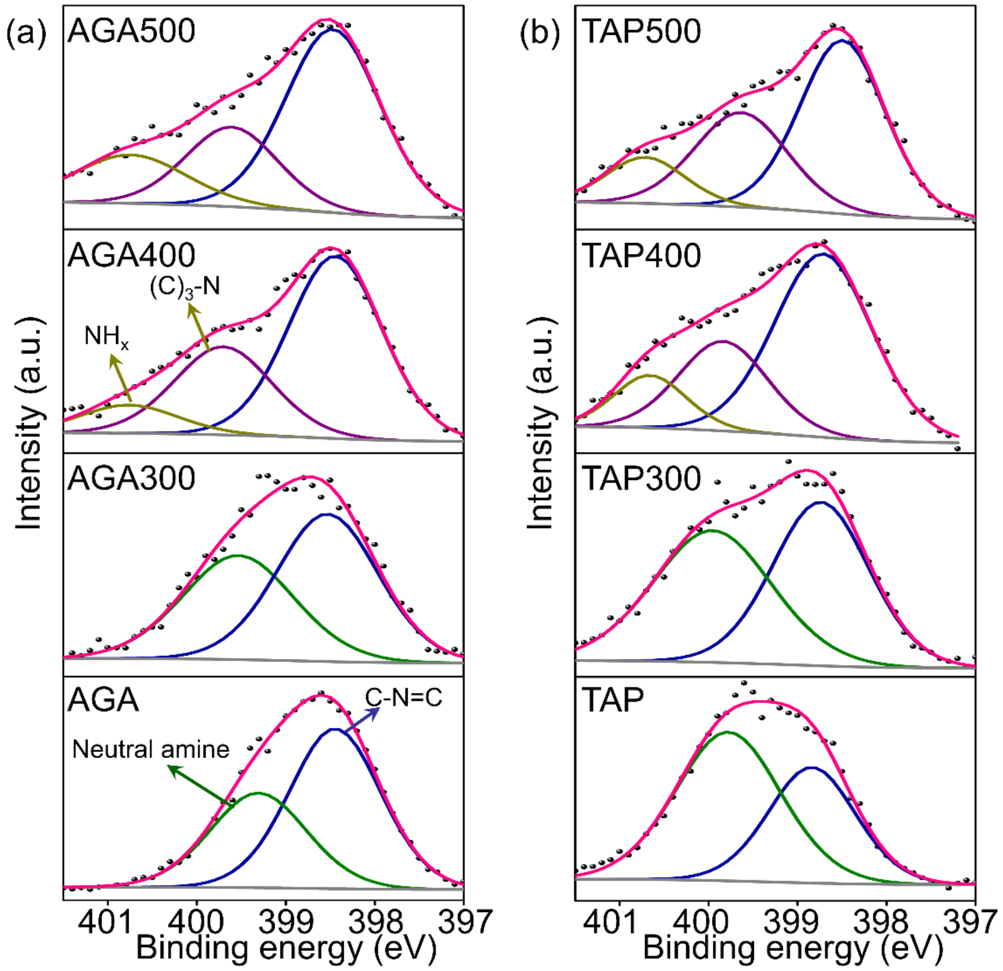

Figure S14. High-resolution N 1s XPS spectra of (a) AGA- and (b) TAP-derived materials.

Table S1 C/N molar ratios of AGA- and TAP-derived materials based on XPS results.

Sample	C/N molar ratio	Sample	C/N molar ratio
AGA	1.08	TAP	1.05
AGA300	1.11	TAP300	1.29
AGA400	1.36	TAP400	1.69
AGA500	1.39	TAP500	1.82

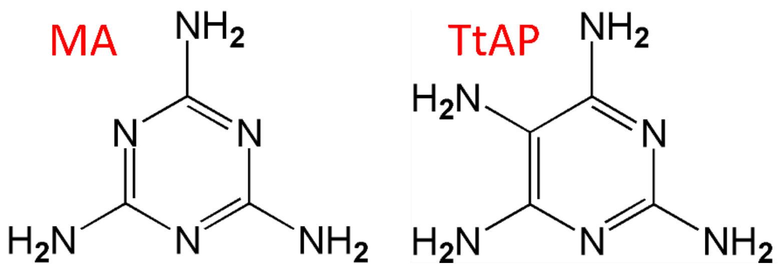

Figure S15. Molecular structures of MA and TtAP.

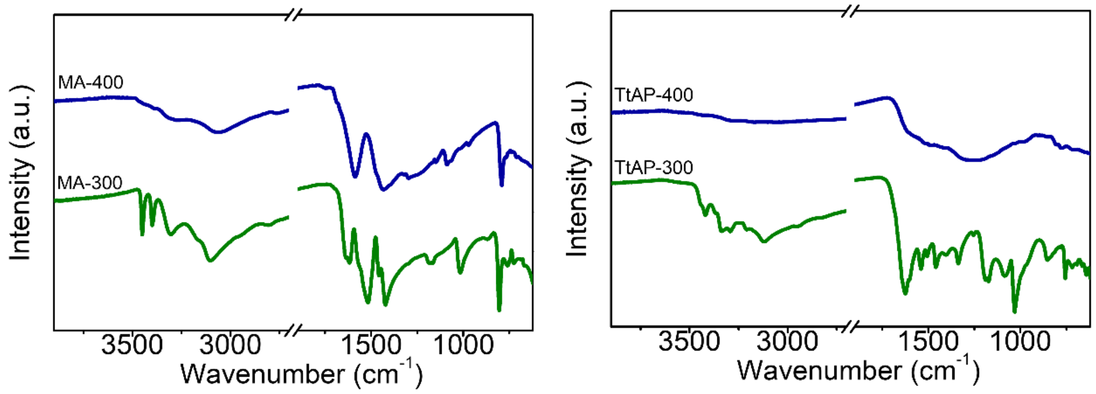

Figure S16. FTIR spectra of MA (a) and TtAP-derived materials (b).

The bands at 2900-3500 cm^{-1} are assigned to N-H stretching, the strong peaks between 1200 and 1700 cm^{-1} are associated with stretching vibrations of triazine rings, and the sharp peak at 806 cm^{-1} corresponds to the breathing vibration of the triazine group. MA300 and TtAP300 still retained the sharp peaks of the amino group. At a calcination temperature of 400 °C, the signals of the amino group begin to broaden and weaken in the MA400 spectrum, and even more so in the TtAP400 spectrum.

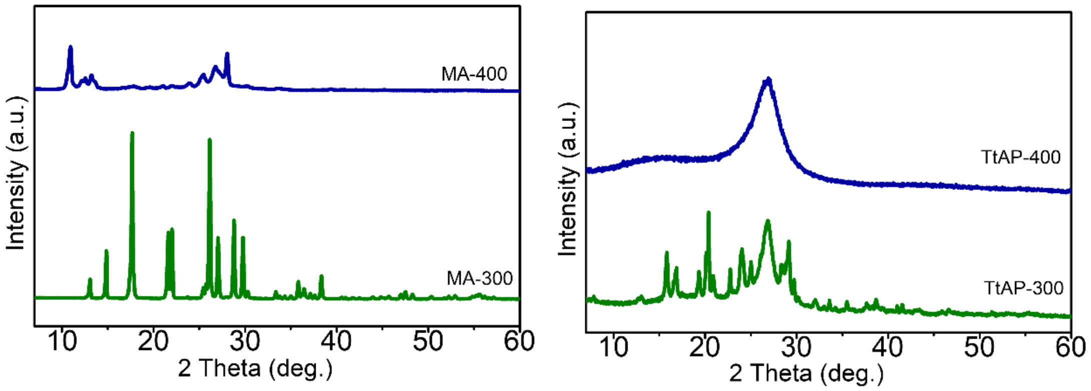

Figure S17. XRD patterns of (a) MA- and (b) TtAP-derived materials.

The diffraction patterns of MA300 and TtAP300 still resembles those of MA and TtAP. At 400 °C, the XRD pattern of MA400 showed obvious changes, the two strongest peaks at 11° and 28° can be attributed to the in-plane and interplanar stacking of a weakly ordered polymeric structure. and TtAP400 showed a (002) peak (at 26.5°) and a broad (100) peak (at 12-15°) of graphitic structures.

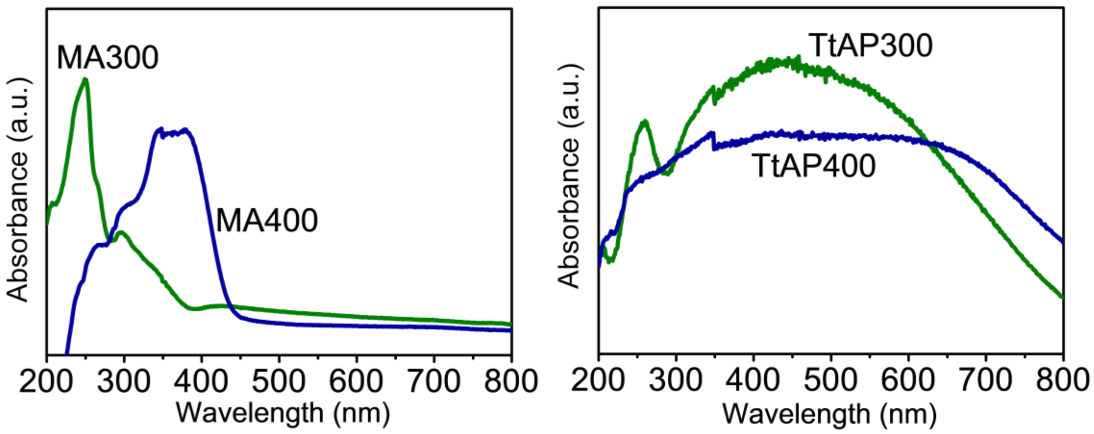

Figure S18. UV-vis spectra of (a) MA- and (b) TtAP-derived materials.

The absorption band edges of both AGA- and TAP-derived materials showed a red shift upon calcination at higher temperatures.

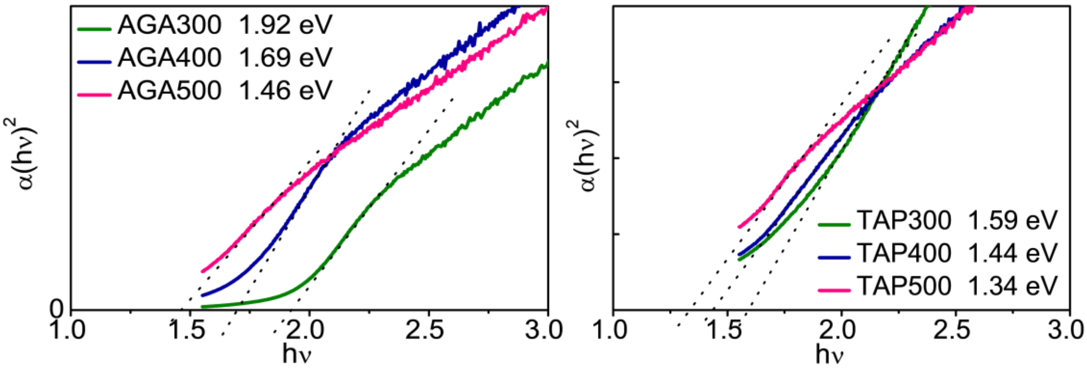

Figure S19. Tauc plot for AGA- and TAP-derived materials.

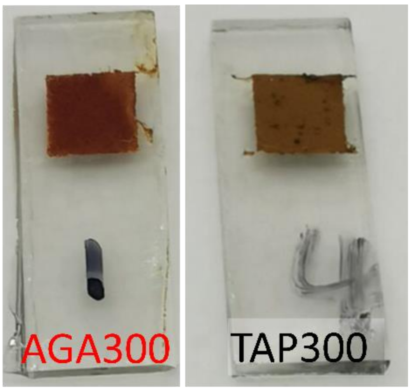

Figure S20. Digital photographs of AGA300 and TAP300 electrodes.

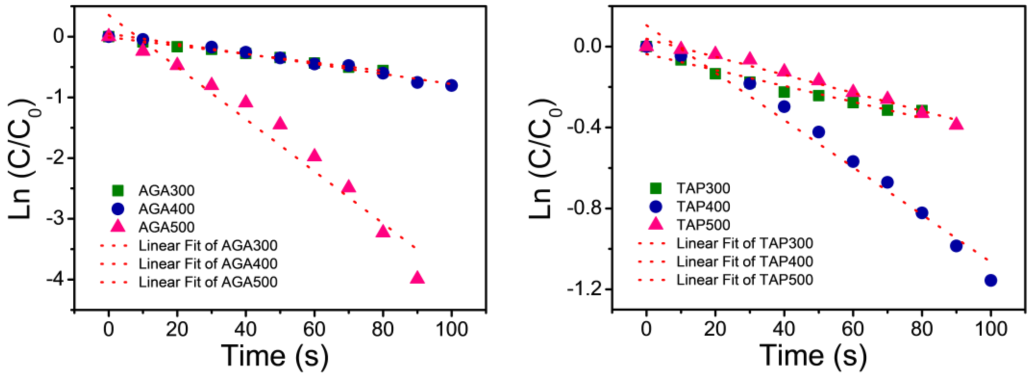

Figure S21. Relative concentration of RhB (natural logarithmic scale) as a function of illumination time for AGA- and TAP-derived materials.

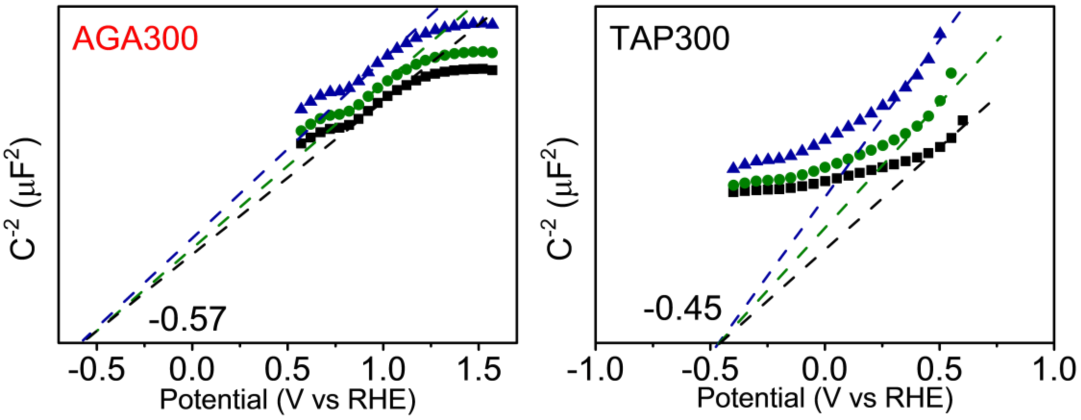

Figure S22. Mott-Schottky measurements of AGA300 and TAP300.
